# Digital and Technology-Enabled Approaches in Dietary Assessment: Addressing Bias, Error, and Feasibility in Population- and Community-Based Research

**DOI:** 10.1016/j.advnut.2026.100667

**Published:** 2026-06-01

**Authors:** Caroline A Joyce, Bess L Caswell, Reina Engle-Stone, Aulo Gelli, Christine P Stewart

**Affiliations:** 1 Institute for Global Nutrition, University of California, Davis, CA, United States; 2 United States Department of Agriculture, Agricultural Research Service, Western Human Nutrition Research Center, Davis, CA, United States; 3 Poverty, Gender, and Inclusion Unit, International Food Policy Research Institute, Washington, DC, United States

**Keywords:** 24-h recall, food record, dietary intake measurement error, portion size estimation, food composition databases, recipe disaggregation, nutrition surveillance, community-based nutrition survey, low- and middle-income countries, technology

## Abstract

Dietary intake data are essential for understanding diet–disease relationships, informing policy, and evaluating nutrition interventions. This is particularly challenging in population- and community-based research, where varying dietary patterns, motivation to participate, and practical constraints limit the feasibility of highly controlled dietary assessment methods. Advances in digital tools, including web- and smartphone-based assessment, image- and speech-based methods, wearable sensors, and artificial intelligence are transforming how dietary data are collected, processed, and analyzed. This narrative review examines how digital and technology-enabled approaches impact key sources of bias and error throughout the stages of dietary assessment, from sample selection and participant reporting to food classification and nutrient composition assignment. We also assess the feasibility of implementing these approaches in large-scale research settings by considering resource requirements, respondent burden, and scalability. Evidence supporting these technologies varies considerably across applications, populations, and settings, and relatively few approaches have been evaluated in large-scale or low-resource research contexts. Although many technologies can reduce specific sources of bias and measurement error, they may also introduce new challenges, such as selection bias and higher development or implementation costs. These trade-offs are especially pronounced in large and heterogeneous study populations. Constraints related to connectivity, device access, and technical capacity may further limit implementation in low-resource settings. While technological innovations offer significant opportunities to improve dietary assessment, selecting the appropriate assessment method requires careful consideration of the trade-offs between accuracy and feasibility in the intended research context.

**Statement of significance**

This review provides a synthesis of technological approaches to dietary data collection, processing, and analysis, evaluating their implications for bias, measurement error, and feasibility across population- and community-based research.

## Background

Rates of overweight, obesity, and diet-related noncommunicable diseases are rising globally, whereas inadequate micronutrient intake continues to account for a significant burden of disease [1]. Addressing these challenges requires accurate and representative dietary data to identify populations at risk of inadequate nutrient intake, evaluate interventions, and guide policy and resource allocation [2]. Generating such data at scale requires methods that balance precision with respondent burden, cost, and scalability. These needs are especially pronounced in population- and community-based research, where data must be collected across diverse populations with varying levels of literacy, motivation, and access to resources.

Food frequency questionnaires (FFQs), 24-h recalls, and food records remain the primary methods for estimating dietary intake [3]. Each involves trade-offs, particularly in their susceptibility to different sources of bias and error, including selection bias, interviewer effects, social desirability bias, reactivity, and recall limitations [[3], [4], [5], [6]]. Additional measurement errors arise during portion size estimation, food classification, recipe disaggregation, and food composition assignment. Although some of these are inherent to the instruments themselves, others arise from study design, implementation, and contextual factors, such as cultural norms around food reporting, literacy and language barriers, and differences in food environments. Participant burden further constrains data quality by influencing response rates, completeness, and loss to follow-up [[7], [8], [9]]. No single method fully balances accuracy, cost, and feasibility for large samples and diverse populations.

Advances in mobile technologies, digital data collection platforms, and artificial intelligence (AI) have expanded how dietary data can be collected, processed, and analyzed [10,11]. In this review, “technological innovations” refers to digital tools and systems that modify these processes, including both novel approaches and the adaptation and scaling of existing methods (e.g., telephone-based surveys or computer-assisted interviews) enabled by improvements in connectivity, computing power, and data infrastructure. Although these technologies are increasingly applied globally, their adoption and scale-up remain uneven, particularly in some low- and middle-income countries (LMICs) and other low-resource settings, where limitations in infrastructure, internet and smartphone access, trained personnel, and financial resources shape feasibility and performance. When integrated with traditional dietary assessment approaches, technological innovations may reduce bias and/or error, improve efficiency, and lower respondent burden. However, they may also introduce new challenges related to selection bias, data processing, and resource constraints.

Prior reviews have examined technological innovations in dietary assessment, often focusing on specific tools, analytic performance, or applications in high-resource settings [[11], [12], [13], [14], [15], [16], [17], [18], [19]]. Although these provide valuable insights, they rarely evaluate how technologies interact with established sources of bias and error across the full assessment process. In addition, feasibility considerations such as cost, infrastructure requirements, scalability, and applicability across diverse settings are often addressed inconsistently or in isolation from measurement error.

This paper reviews technological approaches in dietary assessment and evaluates their implications for bias and error across the stages of dietary data collection, processing, and analysis. We also considered feasibility and implementation trade-offs, including cost, infrastructure requirements, and scalability. We focused on applications in population- and community-based research, including national surveys, cohort studies, and program evaluations (i.e., studies designed to assess the implementation and impact of nutrition interventions, ranging from community-level to large-scale programmatic evaluations). These study designs differ from controlled or clinical feeding studies in that they generally involve more heterogeneous populations, less restrictive inclusion criteria, and less controlled data collection environments.

**FIGURE 1:**
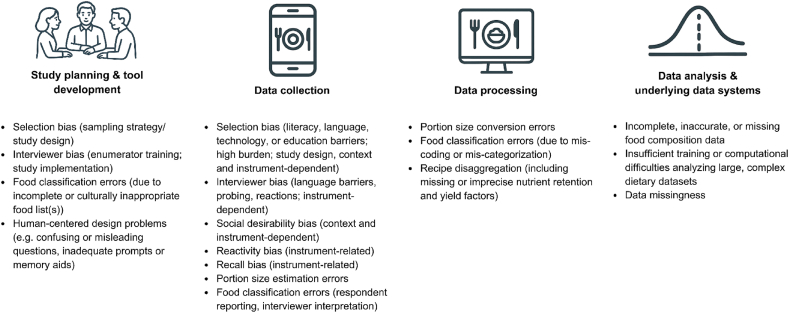
Sources of bias and error across stages of dietary assessment. Biases may originate from dietary assessment instruments, study design and implementation, or broader contextual factors. Errors arise at multiple stages of the dietary assessment process, including study design; data collection, processing, analysis; or underlying data systems. These sources are interrelated and may compound in population- and community-based research settings.

First, we outline major sources of bias and error in dietary assessment (Figure 1), distinguishing between those arising from assessment instruments and those related to study design and implementation. We then examine how emerging technologies influence these sources of bias and error in common dietary assessment methods (Table 1), highlighting both potential improvements and new challenges. Finally, we assess feasibility and implementation trade-offs, with particular attention to constraints in low-resource settings.

**TABLE 1:**
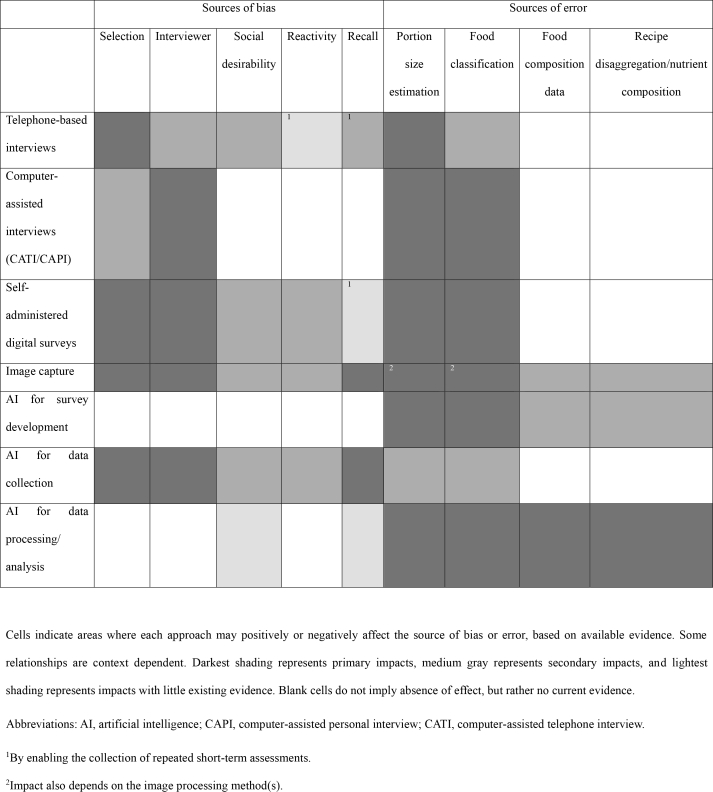
Mapping of technological approaches to sources of bias and error in dietary assessment

### Literature identification and selection

This narrative review is based on a targeted search of PubMed and Google Scholar, supplemented by reference list screening. Searches used terms related to dietary assessment methods, technology-enabled approaches (e.g., mobile, computer-assisted, image-based, AI), and study context (e.g., population- and community-based research, LMICs). We included peer-reviewed and relevant gray literature describing the development, validation, or application of these approaches where available, with emphasis on nonclinical settings. Articles were selected iteratively based on relevance to bias, error, feasibility, and implementation.

### Sources of bias in dietary assessment

Sources of bias, or systematic errors, in dietary data arise from multiple domains. Some reflect study design and implementation, including sampling strategies, survey administration, and interviewer characteristics. Others are inherent to the assessment instrument, for example, reliance on memory or portion size estimation challenges. Additional influences arise from contextual factors, such as cultural norms, literacy, and food environments, which shape both reporting and participation. Although these domains are conceptually distinct, they often interact in practice. In the following sections, we highlight major sources of bias and indicate the primary domain(s) through which they arise.

#### Selection bias

Selection bias is a potential concern for all dietary assessment methods, as participation depends on access, capacity, and willingness to respond. It arises primarily from study design and implementation, particularly sampling strategies and barriers to participation, though it may be exacerbated by features of specific assessment tools. The absence of survey materials or enumerators in all relevant languages increases the risk of excluding populations across most data collection methods [3,20]. Self-administered surveys may further exclude individuals with low literacy or educational attainment, or certain disabilities—groups which are often underrepresented in nutrition research [3,18,20,21]. Respondent burden is also a key driver of selection bias and loss to follow-up, as longer or more complex instruments, such as food records and detailed FFQs, require sustained attention and motivation [3].

#### Interviewer bias

Interviewer bias arises from study implementation and is specific to interviewer-administered methods. Differences in how interviewers ask questions, probe for information, react to participants’ food choices, or document responses—due to linguistic or cultural barriers, inadequate training, or limited knowledge of local foods—can create random or systematic error [5,6]. Although interviewer bias can be minimized through training and quality control or by randomly assigning interviewers across geographical areas, days of the week, or households, it cannot be fully eliminated [5,6]. Some data collection tools, however, are less susceptible to interviewer bias, depending on the extent to which the instrument uses standardized prompts and probing [3].

#### Social desirability bias

Social desirability bias occurs when survey respondents report foods, beverages, or supplements incorrectly due to perceived judgement by the data collector or research team, reflecting an interaction between respondent behavior and study context [5]. Participants tend to underreport foods they believe are undesirable and overreport foods they believe are desirable, usually resulting in underestimates of energy intake and overestimates of some micronutrients [3,5]; however, the extent and direction of misreporting can vary across cultures, respondent characteristics, and assessment methods [5,22,23].

#### Reactivity bias

Awareness of being observed or recorded can alter the types and quantities of foods consumed, particularly during food records and prescheduled 24-h recalls [3]. Reactivity bias is primarily linked to the assessment method (e.g., prospective recording) but may be influenced by study context and participant awareness of observation. In self-reported food records, reactivity bias often increases as the burden of data collection increases [3,24]. Like social desirability bias, it generally skews consumption toward healthier foods and smaller quantities, leading to underestimates of energy intake and overestimates of micronutrient intake [3]. Reactivity bias may also vary systematically by participant characteristics [3,24].

#### Recall bias

Recall bias, inherent in retrospective dietary assessment methods, reflects respondents’ cognitive and behavioral limitations in remembering the types and quantities of foods, beverages, and supplements consumed [5,6,25]. Errors may arise from omissions (items consumed but not reported), intrusions (items reported but not consumed), or incorrectly recalling portion sizes, which can introduce random or systematic error [6]. Snacks, condiments, and beverages are often forgotten, whereas portion sizes may be consistently overestimated or underestimated [5,6]. Less motivated participants may forget to report foods due to survey fatigue, leading to underestimates of nutrient intakes [3].

### Sources of error in dietary assessment

In contrast to bias (systematic error), the following sections focus on sources of random error, reflecting variability in dietary estimates that is not consistently directional, though in some cases such errors may show systematic patterns (e.g., by food type or population subgroup). Errors in dietary intake estimation arise across multiple stages of the assessment process. Some originate from respondent reporting and measurement (e.g., portion size estimation), whereas others are introduced during data processing and analysis, including food classification, recipe disaggregation, and assignment of nutrient composition values. Additional sources of error reflect limitations in underlying data systems, such as incomplete or variable food composition data. These errors may compound, particularly in large-scale studies where standardized assumptions are applied across diverse foods, preparation methods, and populations. In the sections below, we describe major sources of error and indicate the primary stage at which they arise.

#### Portion size estimation

Portion size estimation errors arise from both respondent-related factors (measurement and reporting) and researcher decisions about reporting tools and survey response options. This differs from recall bias in that it reflects limitations in respondents’ cognitive ability to estimate volumes and weights, and can therefore affect both retrospective and prospective dietary assessment methods [6]. Estimation is especially difficult in FFQs when foods are consumed as part of mixed dishes, or when grouped together despite being consumed in different frequencies or quantities [3,6,26]. Twenty-four-hour recalls generally require less cognitive load and yield more precise estimates, but they require substantial preparatory work to identify culturally appropriate portion size estimation aids [3,6,27,28].

Additional errors may be introduced during data processing when converting reported portion sizes to standardized units (e.g., grams) using predefined conversion factors [28]. Standard units and food atlases typically correspond to weights estimated in advance of the survey, whereas household utensils require volume and density conversions for each reported food [28]. Tableware poses an additional challenge, as dishes and utensils can hold food quantities that exceed their level-full volume. To account for this, food- and utensil-specific “heaping factors” are needed, which may introduce random or systematic error [11,28].

#### Food classification

Food classification errors arise at multiple stages of dietary assessment, including respondent reporting, interviewer interpretation, and data processing during coding and categorization. Participants may misreport foods; fail to recall or report sufficient details about a given food, beverage, or supplement; or not know specific ingredients or preparation methods of mixed dishes. Subsequently, interviewers may misinterpret or incorrectly record responses, and analysts can miscode entries during data processing.

Survey design can introduce further errors. For example, electronic food records, 24-h recall surveys, and FFQs often use predetermined food lists and food group filters, which must be tailored to the study population [4,29]. Although exhaustive lists reduce misclassification, they increase respondent and interviewer burden. Longer lists may even increase bias, because survey respondents tend to select foods near the beginning of the list, even if a better match is available further down [30]. Some data collection tools use a search field, but this approach can be hindered by spelling discrepancies or variations in food names by geographical region or language [20,21].

FFQs are particularly prone to misclassification because they rarely capture details of foods consumed, such as the cooking method [3]. Similar errors can occur in food records and 24-h recalls when the descriptions of “other” foods and beverages (i.e., those not included in the food list) are not sufficiently thorough for reclassification during analysis [6]. Misclassification can cause overestimation or underestimation of nutrient intakes. These errors may be random, or they may introduce bias if they vary systematically by food type, preparation method, or participant characteristics [3].

#### Food composition data

Errors related to food composition data arise primarily from limitations in underlying data systems. Historically, food composition tables have provided a single nutrient profile for each listed species of food, sometimes distinguishing anatomical parts (e.g., cuts of meat), reflecting a representative nutrient composition derived from available samples [31,32]. This approach is practical when data are scarce, which is often the case, especially in LMICs [4,31,33]. In reality, nutrient composition varies by cultivar, geography, season, fortification, and processing and storage methods [4,31,34]. Animal-source foods vary similarly, reflecting differences in feed and production systems [33]. Moreover, thousands of edible wild and indigenous crops, insects, and animals lack nutrient composition data altogether [33,35]. When values are missing, researchers must substitute data from similar foods, introducing assumptions and potential bias [4].

For processed foods, aggregate values are often used to represent numerous branded products. Dietary supplements present similar challenges: nutrient content varies by brand, dose, and formulation, and comprehensive, up-to-date supplement composition databases are not consistently available across countries. Substituting data from other countries or applying composite values for foods and supplements therefore introduces bias [4,31,34]. Food composition data are further constrained by lack of detail on culturally and geographically specific preparation methods, fortification practices, the rapid introduction of new products, and variations in analytical techniques [4,31].

#### Nutrient composition of recipes

Estimating the nutrient composition of mixed dishes and cooked variations of individual foods (hereafter “recipes”) introduces additional errors during data processing, particularly when assumptions are required about ingredients, preparation methods, and nutrient retention. Food composition databases provide data for raw, edible portions of individual food items, with little, if any, data for cooked or prepared foods [36]. When recipe values do exist, they often represent an average or “typical” preparation method across the country [5,37]. Because direct laboratory analysis is impractical for large-scale surveys [38,39], nutrient values are estimated arithmetically using ingredient names, raw weights, nutrient values, cooking methods, nutrient retention factors, and yield factors (or the final prepared weight of the recipe) [40,41].

Nutrient retention factors are available at the food group level for a limited set of cooking methods [[42], [43], [44]]. Although nutrient losses vary substantially among cooking methods, food groups, and parts of the same food (e.g., cuts of meat), retention factors rarely account for these variables [42,43,45]. Yield factors account for absorption or loss of water, fat, alcohol, and/or salt during cooking [46]. They are usually applied at the recipe level, but can also vary widely depending on the combination of ingredients, cooking method, and cooking duration [41,46]. Current methods may reasonably estimate the macronutrient composition of recipes, but often introduce significant errors for micronutrients, especially those present in small quantities or vulnerable to degradation during cooking [38,39,45].

### Leveraging technology to reduce bias and error

Although measurement errors are generally localized to specific stages of the dietary assessment process, sources of bias are often cross-cutting and may arise from multiple domains, including the assessment instrument, study design, and context. Technological approaches can address bias and error during data collection, processing, and analysis, but their effectiveness depends on the stage and context in which they are applied. Much of the available evidence for these approaches comes from high-income settings, with limited data from low-resource settings. The following sections describe each technology, its implications for bias and error, and feasibility considerations, organized by stage of the dietary assessment process.

#### Telephone-based interviews

Telephone-based interviews are interviewer-administered dietary assessments, most commonly 24-h recalls or FFQs, conducted remotely via mobile or landline phones. They are widely used in high-income settings (e.g., the United States NHANES), but less commonly implemented in LMICs, although mobile phone ownership is increasing rapidly among women and lower-income populations in LMICs [47].

Telephone-based approaches primarily affect selection bias due to differences in phone ownership, connectivity, and the likelihood of answering calls. These factors vary by sex, socioeconomic status, and location, particularly in low-income settings where disparities in phone ownership persist [[47], [48]]. For example, in rural Kenya, nonresponse was threefold higher for phone-based dietary surveys (19%) than in-person surveys (6%), despite in-person recruitment and intra-household phone sharing [48]. Even in high-income settings, nonresponse can be substantial; in the 2011–2012 Australian National Nutrition and Physical Activity Survey, only 64% of eligible participants completed a second recall by phone following an in-person interview [49]. Telephone-based surveys may reduce some barriers to participation (e.g., not needing to remain at home), which could improve participation in certain groups [13,14], but evidence on the overall effects on representativeness in dietary assessment is limited [14]. Although strategies such as phone sharing, providing devices, and in-person recruitment may improve participation [[50], [51]], these measures may not fully address differential nonresponse.

There is little evidence regarding the impact of telephone interviews on social desirability and interviewer bias. Phone-based surveys may increase reporting bias where trust is low, but reduce it in contexts where anonymity is valued [48]. Mode-related differences appear to depend largely on the demographic characteristics of the respondents [52], survey content, and cultural context, but these effects in dietary surveys remain unclear.

A household consumption survey in urban Ethiopia found that questions asked later in phone interviews were associated with 12% lower reported consumption [53], suggesting higher recall bias, possibly due to respondent fatigue. However, phone-based data collection enables the collection of repeated short-term recalls and other near-real-time approaches, such as ecological momentary assessments, which may reduce reliance on memory compared with longer recall periods [54]. Although these more prospective approaches may introduce reactivity bias, additional evidence is needed to assess the overall effects of these methods on data quality.

Portion size estimation presents a particular challenge for phone-based surveys due to the lack of physical aids (e.g., household utensils, food replicas, moldable and pourable materials, or food atlases) [27]. The absence of these tools was expected to reduce accuracy, but available evidence suggests that portion size estimates obtained via phone interviews may be comparable to those collected in person [48,51,55]. The use of digital food atlases or other visual aids may further support estimation as smartphone and internet access expand [56].

To our knowledge, only one study has directly compared micronutrient intakes from phone and in-person 24-h recalls against a reference standard within the same population [51]. In that study, conducted in rural Sri Lanka, phone-based recalls produced equivalent estimates for protein and 6 micronutrients relative to weighed food records within 15%, whereas no nutrient intake estimates were equivalent for in-person recalls [51]. However, this evidence is limited to a single study and a subset of nutrients.

Telephone-based surveys reduce logistical barriers by eliminating the need for in-person visits, which may lower implementation costs. In the same Sri Lanka study, phone-based 24-h recalls were estimated to cost 27% less than in-person surveys [57]. To our knowledge, no additional studies have directly evaluated the costs or cost-accuracy trade-offs of collecting phone compared with in-person dietary data. Feasibility of telephone-based interviews depends on reliable phone access, network connectivity, and respondent availability. Overall, telephone-based approaches may be scalable in both high- and low-resource settings, but their performance depends on context-specific trade-offs between cost, accessibility, and data quality.

#### Computer-assisted interviews

Computer-assisted personal (CAPI) and telephone (CATI) interviews are digitized, and interviewer-administered dietary assessment methods (i.e., 24-h recalls and FFQs) are conducted using electronic devices such as tablets or computers. Although these approaches are now well established in high-income settings, they are increasingly being adapted and implemented in LMIC contexts.

Features such as multilingual interfaces can reduce selection bias where language barriers would otherwise limit participation, provided that translations to all relevant languages are available. Respondent trust and perceptions of digital data collection may further influence participation. In some contexts, electronic data capture has been associated with increased perceived importance of surveys and improved perceptions of confidentiality relative to paper-based methods [58].

Computer-assisted interviews can reduce interviewer-related bias through standardized question delivery and automated survey flow, for example, skip logic and autocalculation of certain variables [4,58]. These features may also improve the data collection experience for enumerators [58]. However, differential familiarity with digital tools among enumerators or respondents may introduce new sources of bias depending on the research context.

Electronic data collection can reduce several sources of measurement error. Built-in constraints (e.g., required fields and standardized portion size units), automated checks for implausible values, and preprogrammed food lists may reduce data entry errors related to portion size estimation and misclassification [4,58,59]. Real-time data transmission enables centralized monitoring and feedback, reducing missing or inconsistent values [58]. Additional innovations like AI-assisted semantic search may further reduce food classification errors by improving matching during data entry [60].

Evidence from a validation study among women in Vietnam suggests that these tools can achieve comparable accuracy to the traditional pen-and-paper method. In an evaluation of INDDEX24, a computer-assisted dietary recall platform, both digital and paper-based surveys produced estimates within 10% of the weighed food record reference values for energy, macronutrients, and 4 of 5 micronutrients [61]. A comparison of web- and paper-based FFQs in Canada similarly found high levels of agreement in nutrient estimates across modes, although this study assessed reliability rather than validity against reference measures [62].

Computer-assisted interviews often involve higher upfront costs related to survey development, programming, and equipment, but these are likely to be offset by efficiencies in data entry, cleaning, and project administration [58,61,63,64]. In Nepal’s 2011 Demographic and Health Survey, tablet-based data collection reduced the total data collection period by 15% compared with the previous paper-based survey through faster data transfer (5–7 min compared with days for paper forms) and real-time feedback [58]. Cost savings have been documented in both high-income and LMIC settings, particularly when devices are reused across survey rounds or implemented at scale [58,61,64]. CATI may provide additional cost advantages by eliminating enumerator travel and accommodation, which often constitute a large share of survey budgets [48,51].

Feasibility of these approaches depends on infrastructure, including reliable electricity, internet access, device maintenance, and the affordability of mobile data or airtime for respondents. Although electronic data collection can improve efficiency and usability, factors such as battery life, connectivity, and electricity supply may pose challenges in some settings, requiring mitigation strategies such as spare batteries or solar chargers [58]. Even where network and physical infrastructure support CAPI and CATI implementation, the cost of data bundles may still limit participation or survey completion, particularly in LMIC settings. Collaborative development of standardized electronic platforms may further improve efficiency by enabling reuse and adaptation across settings, including incorporation of location-specific food lists, portion size conversion factors, and food composition data [4,65,66].

#### Self-administered digital surveys

Computer-, tablet-, and smartphone application-based instruments can be used to collect self-administered food records, 24-h recalls, or FFQs [16,67]. These are widely used in high-income settings but have been less frequently developed and validated for use in LMICs [21].

Self-administered tools can introduce selection bias due to differences in literacy, language, digital access, and familiarity with technology, particularly in low-income settings where internet access and device ownership remain uneven [18,21,56]. Even in high-income settings, lower accuracy and participation have been observed among children, older adults, and individuals with lower educational attainment or from minority groups [20,[68], [69], [70], [71]]. On the other hand, computer- and app-based methods enable flexible scheduling for participants, which may improve participation in some population groups.

The most significant advantage of self-administered data collection tools is the elimination of interviewer bias. Removing interviewers may also significantly reduce social desirability bias in some contexts, but self-administration may increase respondent burden and reactivity, particularly for repeated or detailed data entry.

As with phone-based interviews, self-administered digital data collection methods enable the implementation of shorter, more frequent reports, including ecological momentary assessments, which may reduce recall bias. In a study among Dutch adults, repeated 2-h recalls collected via a smartphone app underestimated protein and potassium to a lesser extent than self-administered web-based and interviewer-administered telephone-based 24-h recalls (14% compared with 18% for protein and 11% compared with 16% for potassium) [72]. Shorter recall periods and lower respondent fatigue likely contributed to these improvements [72]; however, validation of additional nutrient and in other contexts remains limited.

Self-administered tools rely on respondents to identify foods and enter data without interviewer support, which may increase omission, intrusion, and food classification errors. In a study among United States adults, 66% of foods reported using a self-administered recall method matched observed intake, compared with 75% using an interviewer-administered approach [30]. The self-administered recalls also had higher rates of intrusions. Similar patterns have been observed among American children: 57% of foods matched and 23% of consumed foods were omitted in interviewer-administered recalls, compared with 37% and 35%, respectively, in online self-administered surveys [73]. These results suggest that interviewer probing and familiarity with food lists may improve accuracy, with a larger benefit observed among children than adults. Portion size estimation error is also a concern in self-administered tools, as respondents must estimate quantities without interviewer support. Although interactive aids and digital food atlases may improve estimation, their effectiveness depends on their usability and respondent engagement.

Evidence on the validity of self-administered digital data collection instruments relative to corresponding nondigital methods using a reference standard measurement is scarce. In an Australian study, energy intake did not differ between web-, computer-, and paper-based food records when compared with energy expenditure measured by indirect calorimetry and accelerometry; however, the study did not assess other nutrients [74]. A United Kingdom validation study found nutrient-specific differences relative to biomarkers, with greater underestimation of energy (32% compared with 23%) and sugar (43% compared with 29%) in web-based recalls but smaller overestimation of protein, potassium, and sodium intake compared with interviewer-administered methods [75]. After controlling for energy intake, the differences in protein and potassium overestimation between methods were within 5% [75].

Self-administered digital tools can be implemented at relatively low marginal cost and scaled to large populations, as they reduce the need for trained interviewers and travel. One study reported per-respondent costs as low as 4% of those of in-person interviewer-administered surveys, but the analysis did not account for development, adaptation, validation, or data processing costs [71]. These findings are not directly generalizable to quantitative nutrient intake surveys but suggest potential cost efficiencies at scale.

The feasibility of implementing self-administered dietary assessments depends on access to devices, internet connectivity, and digital literacy, which may limit implementation in some LMIC settings. Although offline functionality and expanding connectivity may improve access over time, evidence from LMIC contexts remains limited [18,21,56].

Early evaluations suggest high acceptability and adherence in selected populations. Studies in the United States and France found that most adults preferred self-administered web-based recalls to interviewer-administered surveys [68,76]. Similarly, among young, healthy Australian women, web- and computer-based food records were rated as less difficult, time-consuming, and inconvenient than paper-based records [74]. One of the first smartphone-based diet tracking applications reported >90% adherence among university students at a time when only 27% of United Kingdom adults owned a smartphone [77]. However, these studies relied on volunteer samples with high motivation and literacy, which are associated with higher data quality [3] and may limit generalizability to broader population-based studies.

Usability is generally high in controlled studies, but challenges with navigation, login, and food search are common [21]. Additional features such as gamification, intuitive interfaces, and automated prompts may further improve usability and adherence, especially with repeated surveys [78,79]. Emerging tools, such as portable weight sensors integrated with mobile applications, may further reduce respondent burden and portion size errors [3,80], but their accuracy and scalability in large-scale studies have not yet been evaluated.

#### Image capture

Image capture refers to the use of photographs to document foods and beverages consumed throughout the day and can be integrated into multiple dietary assessment methods. Image-based approaches (relying solely on photographs) function as prospective food records, whereas image-assisted approaches combine photographic data with respondent-reported information (usually text or audio input) in prospective or retrospective methods [81,82]. Images may be captured passively using wearable devices (e.g., cameras, glasses) or actively using mobile phones or handheld cameras [81,83].

Passive image capture can reduce barriers related to literacy, language, or education, potentially improving inclusivity in some populations [14,18,84]. Privacy and trust concerns may negatively impact participation in some groups, though allowing participants to review or delete images may improve acceptability [82,84,85]. In contrast, active image capture requires participant engagement and training, which may introduce selection bias by age, gender, or degree of motivation [14,86].

Although the absence of enumerators eliminates interviewer bias and may reduce social desirability bias, reactivity bias remains a significant concern [3,87]. Participants may alter their behavior when being recorded, particularly with wearable devices or prompted recording [87,88]. Supplementary data collection (e.g., voice or written annotations) may further increase reactivity; in one study among Chinese adults, nearly three-quarters of participants reported that reminders to record voice-annotated videos altered their consumption [88]. Written annotations may be more acceptable but can introduce selection bias in low-literacy populations.

By capturing intake prospectively, image-based methods reduce reliance on memory and associated recall bias, whereas images used in image-assisted approaches can help improve recall during retrospective reporting [3,18,89]. In a study in New Zealand, image-assisted 24-h recalls underestimated energy intake by 8% compared with 15% for traditional recalls relative to doubly labeled water, likely due to fewer omissions and misreporting errors [87]. The researchers determined that images were especially useful for capturing forgotten snacks, including fruits and vegetables, condiments, and beverages, which were the primary drivers of underestimated energy intakes [87].

Both passive and active image capture methods can potentially improve food classification of individual food items, but it remains a significant challenge for mixed dishes [90]. Image-assisted methods may outperform image-based approaches in these contexts by incorporating respondent input [82,91], but variability in the accuracy and level of detail in respondents’ annotations can introduce additional classification error [82].

Accuracy also depends on image quality and downstream processing, including food recognition and portion size estimation algorithms [92]. Embedded tutorials, automated quality checks, and real-time feedback may improve data quality in active image-capture methods, but incomplete image capture can lead to missing data [82,93]. In a small feasibility study among United States adolescents, fewer than half of participants followed all reporting instructions, and only 21% of the reported eating events included before and after photos with the required fiducial marker and text description [94].

Passive capture methods may produce more complete data by reducing reliance on participant compliance and capturing entire eating episodes [81,95]. Additionally, cameras fixed near food preparation areas could reduce errors related to estimating the nutrient composition of recipes by capturing images of all ingredients and quantities used, the duration of cooking, and amount of water used. Image capture for recipe data collection could significantly reduce respondent burden and time to complete interviews when household-level recipe data are needed, but there is little evidence supporting the use of this approach. Moreover, continuous imaging tools generate large volumes of data that increase analytic complexity.

Image-based approaches may reduce enumerator costs and, in some cases, respondent burden, particularly with passive capture [11]. However, wearable devices are typically loaned to participants during research studies, limiting scalability due to cost and logistics. Active capture using participant-owned devices is more feasible but requires training, adherence, and device access [93]. Device constraints, including battery life, storage capacity, and offline functionality, may also affect implementation, particularly in low-resource settings [18,84].

Image-based and some image-assisted data collection methods reduce enumerator training and salary costs but do not necessarily reduce overall survey expenses. Poor-quality images increase data cleaning requirements [14,96], and image-recognition systems require significant upfront investment in model development, training, and validation, even if marginal costs decline with reuse and at scale [97]. In LMICs, training datasets may need to be developed largely from scratch, increasing initial costs and technical requirements. To our knowledge, no studies have comprehensively compared the costs of image-based or image-assisted dietary assessment methods to traditional approaches across data collection and analysis [84].

#### Artificial intelligence

##### AI for survey development

AI can support the development of dietary assessment instruments by generating and organizing key inputs, including food lists, food atlases, food composition data, and recipe databases used across data collection methods [60,98]. By incorporating more diverse, context-specific foods, these approaches may improve the completeness and representativeness of dietary assessment tools. However, their effectiveness depends on the underlying data and may be limited if certain populations or food environments are underrepresented.

AI techniques can also improve food classification and standardization. Web scraping combined with natural language processing can compile food names and recipe information from online sources [[99], [100], [101]], whereas knowledge graphs and AI-assisted semantic search can enhance linking and interpretation of search terms (e.g., “soda” compared with “soft drink”). Machine learning models can automate food group assignments, reducing manual coding requirements; for example, a semiautomated system achieved 89% accuracy in classifying foods within the FoodEx2 scheme [102]. Together, these approaches support more consistent and scalable food classification, while enabling context-specific adaptations. Some AI-assisted features, such as semantic search and improved matching of local food names or synonyms, may also improve respondent usability and reduce data entry burden, while others, such as FoodEx2, are primarily designed for backend coding and harmonization rather than direct respondent interaction.

Convolutional neural networks, a type of machine learning designed to analyze visual data, can similarly be used to organize image repositories for food atlases [98]. Images of locally available foods preloaded into survey tools may reduce misclassification errors by helping respondents identify and report foods more accurately [5].

AI-enabled tools may improve efficiency and scalability in survey development by reducing the time required to compile and classify food lists, food composition data, and recipe databases. However, implementation depends on access to large, high-quality datasets and technical capacity for model development and maintenance. In settings where locally specific foods are not well represented in existing datasets, additional data collection and model training may be required. Incorporating AI-generated food lists and atlases into survey platforms may also improve usability through more intuitive search and navigation, potentially reducing respondent burden and data entry time [103].

##### AI for data collection

AI can be integrated into computer- and app-based dietary assessment methods to support data collection through automated interviewing, multimodal input (text, voice), and context-aware data capture. AI interviewers, AI-enhanced interactive voice response (IVR) systems, and chatbots are increasingly being used to replace interviewers entirely in telephone-, mobile app-, and web-based surveys [[104], [105], [106]]. AI interviewers and AI-enhanced IVR differ in that these systems can process verbal responses, whereas traditional IVR systems are less flexible, relying on keypad responses or limited voice response menus [107]. Chatbots range in their flexibility but are confined to text-based exchanges [107].

AI-enabled data collection may reduce selection bias by lowering literacy and language barriers through verbal or multimodal input. However, modality choice can introduce new biases: although text-based input may be more acceptable in public settings, it may exclude respondents with limited literacy, whereas verbal input may raise privacy concerns. A systematic review found similar levels of underreporting in verbally reported dietary data compared with traditional methods, though discomfort with speaking aloud was reported in some studies [108].

These data collection tools may also introduce selection bias related to access to mobile devices, connectivity, familiarity with digital interfaces, privacy and data security concerns [63,109]. Evidence from LMICs suggests that these barriers can be substantial. In a study across 6 LMICs, response rates for IVR-based surveys were 3%–10% after 3–4 contact attempts using a combination of IVR and SMS, compared with 69%–89% for in-person surveys after 1–2 contact attempts [110]. Among women in Uganda, 25% of participants were lost to follow-up in an IVR-based dietary survey due to technical challenges, including poor network connectivity [111].

In AI-enhanced data collection methods, deep learning and natural language processing improve speech recognition, transcription, and translation and can standardize survey question delivery, thereby reducing interviewer bias [101,112]. The absence of an interviewer may also reduce social desirability bias, but concerns related to privacy, trust, and data security may influence reporting behavior [63,109].

AI applications may reduce recall bias and food classification errors through context-aware data capture [90,113]. For example, passive data sources such as location, Wi-Fi signals, or digital receipts can supplement self-reported intake and improve accuracy [90,113]. In a pilot study among United States young adults, incorporating contextual sensor data (location, physical activity, and ambient sound) increased agreement between foods reported in recalls and ecological momentary assessments by 5.6% [113]. In Uganda, IVR-based 24-h recalls captured population-level diet quality indicators with moderate agreement relative to interviewer-administered recalls, but respondents tended to underreport the number of foods consumed, indicating higher omission errors with IVR [111]. Additional research evaluating the validity of these methods is needed.

Augmented reality (AR) tools may improve portion size estimation by allowing respondents to visually match consumed portions using overlaid 3-dimensional food models rather than relying on abstract size descriptors or memory alone [114]. Repositories of 3-dimensional food models can be generated relatively efficiently using photogrammetry [114], reducing the need for survey teams to manually compile conversion factors for every food-unit combination.

Evidence supporting the use of AR applications dietary assessment has largely focused on prospective data collection and has rarely been validated against traditional data collection methods [[114], [115], [116]]. In a small pilot study, one AR application significantly overestimated energy and macronutrient intakes relative to observed weighed records, despite estimating gram-level portion sizes accurately [115]. Another study reported lower estimation error compared with unaided methods or the use of hand-based size references but relied on smartphones equipped with depth sensors and controlled conditions [116]. Evidence for the use of AR-based tools in retrospective data collection is sparse, with 1 study finding no clear advantages over traditional portion size aids in estimating nutrient intakes [117].

AI-enabled data collection tools may reduce reliance on trained interviewers and improve scalability, but feasibility varies by approach. IVR systems may be more feasible in low-resource settings due to lower cost and technical requirements compared with AI interviewers [118], though evidence on feasibility, acceptability, and cost-effectiveness in dietary assessment remains limited. Implementation depends on infrastructure, including mobile device access, network connectivity, and data security systems. Low response rates and loss to follow-up in LMIC studies highlight the importance of these constraints [110,111]. More advanced approaches, such as context-aware systems and AR, require substantial technical capacity, high-quality data integration, and, in some cases, specialized hardware, which may limit scalability in large-scale or low-resource settings.

##### AI for data processing and analysis

During data processing and analysis, AI can automate tasks such as portion size estimation, food classification, and linkage to food composition data. These approaches may reduce bias arising from inconsistent coding and subjective interpretation of dietary data. As with the use of AI for survey development, performance depends on the representativeness of the underlying data and may introduce systematic error if certain foods or preparation methods are underrepresented.

AI applications may also reduce specific sources of measurement error, although performance varies by context. For portion size estimation, machine learning models using image data and probabilistic matching can estimate food weights with relatively low error under controlled conditions (e.g., 4%–5% mean error for rice and chicken) [119,120]. However, accuracy declines in population- and community-based settings where images vary in angle, lighting, and calibration, and foods differ in density or preparation method [11,119]. Additional manual input, context-specific training, and/or model adaptation is often required, particularly for culturally specific or mixed dishes [119].

For food classification, machine learning and natural language processing systems have demonstrated moderate to high accuracy. A semiautomated system developed by the European Food Safety Authority achieved ∼80% accuracy in assigning food categories from text-based descriptions [102], and speech-to-text systems applied to dietary records have reported accuracies exceeding 80% [108]. Given the large number of food codes and classification levels in systems such as FoodEx2, achieving this level of agreement suggests that semiautomated tools may meaningfully reduce manual coding, even if some degree of human review remains necessary.

Computer vision and deep neural networks, a machine learning approach used for pattern recognition, can classify foods from images with reported accuracies generally exceeding 70%–80% [[121], [122], [123]]. However, performance depends on image quality, training data, and food type, and these models remain limited in identifying less visible characteristics. Mixed dishes present particular challenges, often requiring a combination of automated recognition and respondent input [99,124]. Context-aware models that incorporate data such as time, location, or respondent characteristics may further improve classification [119,121,122].

Machine learning models have been proposed to identify and adjust reporting bias (e.g., social desirability and/or recall bias) using participant characteristics and reference data. This approach is similar to regression calibration, the established method for correcting measurement error in dietary assessment [125], but offers potential advantages, such as ranking predictors and capturing nonlinear relationships [126]. To date, few studies have applied machine learning to explicitly address reporting bias in dietary data. In 1 study, a random-forest-based model achieved 78%–92% accuracy in correcting underreported FFQ items [126]. In principle, similar techniques could extend to 24-h recalls, where reporting bias is a persistent challenge, particularly in unblinded intervention studies [127].

AI-based processing can improve efficiency by reducing manual coding, but implementation requires substantial technical capacity, including access to large, high-quality training datasets and computational resources. Neural networks used in dietary assessment are trained using supervised learning, which relies on manually annotated image or text data, or unsupervised learning, which relies on unlabeled data [128]. Supervised models are currently more accurate, with food image recognition studies commonly reporting classification accuracies of 70%–85% under controlled conditions [122,123]. However, these models are resource intensive to develop, especially in settings where locally specific foods may not be well represented [128]. Unsupervised methods have shown promise for feature extraction and depth estimation but have not been widely validated in community-based settings [128,129]. In particular, evidence is needed to support the use of such models in contexts where food is consumed from a shared plate—a common practice in many LMICs.

An AI-based food recognition and processing application, PlantVillage Food Recognition Assistance and Nudging Insights (FRANI), has demonstrated promising results in LMIC contexts. PlantVillage FRANI combines the use of convoluted neural networks for semantic segmentation with user input to complement AI-predicted classification and portion estimation. Among adolescent girls and women in Ghana, the app produced estimates of energy and several micronutrients within narrower equivalence bounds than traditional 24-h recalls when compared with weighed food records [130,131]. Similar results were found in adolescent girls in Sri Lanka and Vietnam, where FRANI performance was at least comparable to 24-h recall, though there was variation in performance metrics across countries [132,133], highlighting the importance of context-specific validation. Validation work is underway to examine the use of large-language models to complement semantic segmentation.

Some systems map free-text or spoken food descriptions directly to food composition databases [134,135]. In a small pilot study, one such model yielded estimates of energy and macronutrient intake that were not statistically different from interviewer-administered 24-h recalls [135]. Although early evidence suggests improved efficiency with comparable accuracy, performance, and implementation costs across diverse settings is unclear.

Advances in food composition databases may further support AI-based processing. Efforts to develop standardized, globally applicable food composition databases and expand coverage to include diverse foods and bioactive compounds could improve automated food matching and nutrient estimation [[136], [137], [138], [139]]. Complementary efforts to standardize food description and indexing systems, such as the LanguaL framework developed through the European Food Information Resource Network of Excellence, are underway and may further improve the performance of automated food-linking and classification tools [140]. However, the utility of these systems depends on the quality, completeness, and transparency of the data being collected and compiled [31,137].

When nutrient composition values are missing, AI-based predictive modeling may supplement existing databases, though these approaches require sufficiently large and representative training datasets to produce reliable estimates [31,141]. Additional tools, such as barcode scanning and integration of location-specific fortification or recipe data, may further improve the accuracy and applicability of nutrient estimates in diverse settings [31,142].

##### AI limitations

Many current AI tools require substantial technical capacity, computing power, and high-quality training data, which currently limits their implementation in large-scale or low-resource settings [143]. As a result, approaches that rely on structured text data or lower computational requirements (e.g., IVR or automated classification) may be more immediately scalable.

To our knowledge, no studies have reported the full lifecycle cost or cost-efficiency of implementing AI for dietary assessment, and such evidence is needed to determine whether gains in accuracy justify the development and computational costs. AI also has environmental impacts, consuming water, energy, and minerals, and generating greenhouse gas emissions and electronic waste [144]. Although advances may improve efficiency [145], the net environmental footprint remains unclear [144]. In some cases, AI-enabled methods (e.g., replacing in-person data collection with telephone- or web-based surveys) may offset its impacts by reducing travel and field logistics, but further evidence is needed.

These limitations underscore that the potential benefits of AI depend on investment in data infrastructure; transparent model development; and consideration of costs, feasibility, and equity across settings. As their use expands, analytical standardization and guidance on acceptable error relative to cost-efficiency will be warranted [128].

In conclusions, improved methods of collecting and analyzing dietary intake data in population- and community-based studies are needed to inform national recommendations, diet-related disease research, and nutrition interventions [4]. Digital and technology-enabled approaches can reduce common sources of bias and error across the stages of dietary assessment, including recall limitations, interviewer effects, and errors in portion size estimation and food classification, while lowering costs [5], but may simultaneously introduce new biases, especially in settings with limited accessibility, digital literacy, or acceptability. Although the costs and technical requirements of AI-driven models may currently outweigh incremental gains in accuracy, these may decline over time [97]. Researchers must therefore carefully weigh potential improvements in bias and error against cost, technical capacity, scalability, interpretability, and the risk of introducing new forms of bias.

Priority next steps include rigorous validation of emerging tools and evaluation of feasibility across contexts. Standardized frameworks or reporting tools could support systematic evaluation of dietary assessment methods and their susceptibility to bias and error, particularly by integrating considerations of technology, feasibility, and context. Stronger collaboration across nutrition, computer science, survey methodology, and global health will be essential to develop, validate, and implement equitable dietary assessment innovations across diverse populations and settings. Additionally, the development of a formal ontology distinguishing core dietary assessment methods from enabling technologies, and situating them within the stages of the assessment process, could further improve conceptual clarity and consistency in terminology across studies.

## Author contributions

The authors’ responsibilities were as follows – CAJ, CPS: designed the research, with input from BLC, AG, and RE-S; CAJ: conducted the research, analyzed the data, and wrote the paper, with input from all coauthors, and primary responsible for the final content; and all authors: read and approved the final manuscript.

## Data availability

Data described in the manuscript, codebook, and analytic code will be made available on request pending application and approval.

## Declaration of generative AI and AI-assisted technologies in the writing process

During the preparation of this work, the authors used ChatGPT (OpenAI, GPT-5.2) to support language editing, sentence restructuring, and clarity of expression. After using this tool, the authors reviewed and edited the content as needed and take full responsibility for the content of the publication.

## Funding

The authors reported no funding received for this study.

## Conflicts of interest

The authors report no conflicts of interest.
